# Minority Breast Cancer Survivors: The Association between Race/Ethnicity, Objective Sleep Disturbances, and Physical and Psychological Symptoms

**DOI:** 10.1155/2014/858403

**Published:** 2014-07-02

**Authors:** Pinky H. Budhrani, Cecile A. Lengacher, Kevin E. Kip, Cindy Tofthagen, Heather Jim

**Affiliations:** ^1^Phyllis F. Cantor Center for Research in Nursing and Patient Care Services, Dana-Farber Cancer Institute, Harvard Cancer Center, Boston, MA 02215, USA; ^2^University of South Florida College of Nursing, Tampa, FL 33612, USA; ^3^H. Lee Moffitt Cancer Center & Research Institute, Tampa, FL 33612, USA

## Abstract

*Background*. Limited research has been conducted on the moderating effect of race/ethnicity on objective sleep disturbances in breast cancer survivors (BCSs). *Objective*. To explore racial/ethnic differences in objective sleep disturbances among BCSs and their relationship with self-reported symptoms. *Intervention/Methods*. Sleep disturbance and symptoms were measured using actigraphy for 72 hours and self-reported questionnaires, respectively, among 79 BCSs. Analysis of covariance, Pearson's correlation, and multivariate regression were used to analyze data. *Results*. Sixty (75.9%) participants listed their ethnicity as white, non-Hispanic and 19 (24.1%) as minority. Total sleep time was 395.9 minutes for white BCSs compared to 330.4 minutes for minority BCSs. Significant correlations were seen between sleep onset latency (SOL) and depression, SOL and fatigue, and sleep efficiency (SE) and fatigue among minority BCSs. Among white BCSs, significant correlations were seen between SE and pain and wake after sleep onset (WASO) and pain. The association between depression and SOL and fatigue and SOL appeared to be stronger in minority BCSs than white BCSs. *Conclusions*. Results indicate that white BCSs slept longer than minority BCSs, and race/ethnicity modified the effect of depression and fatigue on SOL, respectively. *Implications for Practice*. As part of survivorship care, race/ethnicity should be included as an essential component of comprehensive symptom assessments.

## 1. Introduction

Breast cancer is the second most commonly diagnosed cancer in the United States and comprises the largest population of cancer survivors, estimated at 2.9 million women and accounting for 22% of all cancer survivors [1]. Advances in breast cancer screening, diagnosis, and treatment have substantially increased the number of breast cancer survivors (BCSs) and with this have highlighted the importance of survivorship needs. A major concern among BCSs is sleep disturbances. BCSs complain of sleep disturbances before, during, and after the completion of treatment negatively affecting their functional status and quality of life [2, 3].

Past research indicates that the prevalence of sleep disturbances in BCSs is 20% higher in comparison to the general population and 32% higher in comparison to patients with gastrointestinal cancer [4, 5]. With the increasing prevalence of sleep disturbances, a standardized assessment to collect and analyze data on sleep is becoming increasingly vital. Previous studies have evaluated the association between symptoms and objective sleep disturbances in patients with breast cancer [6, 7]. These studies have focused primarily on Caucasian women undergoing treatment with limited research among racial/ethnic minorities [8, 9]. Therefore, the goal of this study was to explore the moderating effect of race/ethnicity on objective sleep disturbances and their relationships with physical (i.e., pain and fatigue) and psychological symptoms (i.e., depression and anxiety) among BCSs, who have completed treatment. The specific aims guiding this paper were to (1) explore racial/ethnic differences in sleep actigraphy parameters among BCSs, (2) explore racial/ethnic differences in the associations between sleep actigraphy parameters with physical (i.e., pain and fatigue) and psychological symptoms (i.e., depression and anxiety), and (3) explore whether these relationships (between objective sleep and self-reported symptoms) are modified by race/ethnicity.

## 2. Conceptual Framework

**Figure 1:**
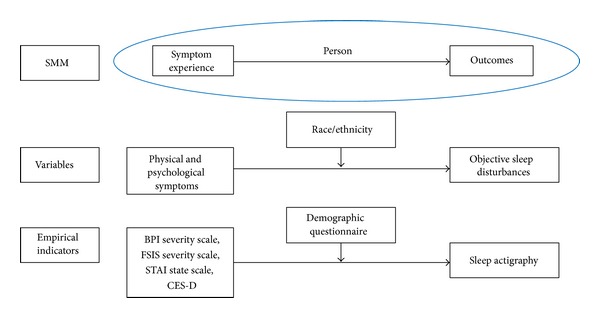
Conceptual framework of the study. The symptom experience was the perception and evaluation of physical and psychological symptoms; the outcome was objective sleep disturbances. This model tested whether the person domain, specifically the demographic variable of race/ethnicity, modified the relationship between the dimensions of symptom experience and outcomes. BPI: Brief Pain Inventory Short Form; CES-D: Center for Epidemiological Studies Depression Scale; FSI: Fatigue Symptom Inventory; SMM: Symptom Management Model; STAI-S: State Trait Anxiety Inventory-State Scale only. Adapted from Humphreys et al. [11].

The conceptual framework for this study (Figure 1) was based on the University of San Francisco School of Nursing Symptom Management Model (SMM) [10]. The SMM illustrates that symptom management is a multidimensional process, which is influenced by the nursing domains of person, health/illness, or environment. The three domains of nursing directly and indirectly impact three dimensions of this model, which include symptom experience, symptom management, and outcomes. Symptom experience is defined as the perception, evaluation, and response to a change in one's usual feeling. Symptom management is a dynamic process with efforts to avert, delay, or minimize the symptom experience [11]. Lastly, outcomes are the consequences of the symptom experience and management strategies.

The SMM is useful for this study of BCSs as sleep disturbances are often presented with other symptoms, such as depression, fatigue, and pain [12]; the SMM dimension of symptom experience takes this into consideration. The symptom experience was the perception and evaluation of study participants' physical (i.e., pain and fatigue) and psychological symptoms (i.e., depression and anxiety) [11]. This experience could vary by BCS age, family history of cancer, and race/ethnicity (person domain), cultural beliefs (environmental domain), and comorbidities, stage of cancer, and type of adjuvant cancer treatment (health/illness domain). The outcome of interest was objective sleep disturbances. Rather than the person domain influencing these dimensions, the proposed model further expanded the SMM by testing whether the person domain, specifically the demographic variable of race/ethnicity, modified the relationship between the dimensions of symptom experience and outcomes.

## 3. Literature Review

Sleep disturbances are increasingly recognized as a side effect of cancer treatment, affecting both physiological functioning and psychological functioning. According to the American Academy of Sleep Medicine [13], sleep disturbances are characterized by difficulty in falling asleep or staying asleep, early morning awakenings, nonrestorative sleep, and daytime sleepiness. Estimates of sleep disturbances vary widely, ranging from 20% to 70% of newly diagnosed or recently treated breast cancer patients, which is twice that found in the general population [14]. Sleep disturbances can persist through treatment and survivorship and are associated with increased physical and psychological symptoms [15].

Epidemiologic studies have shown the importance of sleep disturbances and race/ethnicity as predictors of health and mortality risks [16]. Results from the National Health Interview Study (NHIS) suggested that black ethnicity was a significant predictor of extreme sleep duration (≤5 hours and ≥9 hours). Compared to whites, blacks were less likely to report sleeping for 7 hours. Extreme sleep durations, decreased sleep efficiency, and increased sleep disruptions are commonly associated with comorbidities, early mortality, and poor health among minority women and women with advanced breast cancer [16, 17]. Increased knowledge of the factors associated with sleep disturbances, including the effect of race/ethnicity on sleep disturbances and their association with symptoms, may improve our understanding of the survivorship experience for patients with cancer.

Past research has indicated that minorities were more likely to experience short sleep durations, lower sleep efficiency, and decreased deep wave sleep [18, 19]. Minority BCSs commonly report sleep-related problems that include sleep maintenance (36%), dissatisfaction with sleep (35.3%), difficulty in falling asleep (23.5%), and early morning awakenings (22.4%) [20]. For patients receiving chemotherapy, whites reported more sleep disturbances than black patients [21]. For patients who have completed treatment, blacks were 44% less likely to report sleep disturbances than whites [22]. The reason for these differences in sleep disturbances is questionable and may be explained either by actual biological differences or by a response bias to questionnaires [23]. Research suggests that an inflammation pathway may mediate the association between decreased sleep duration and cardiometabolic diseases, and this pathway may vary in blacks and whites [24, 25]. Response bias refers to positive reframing, repressive coping, or the modification of responses to sleep questionnaires to be perceived as socially desirable [23]. Blacks may cope with sleep problems within a positive self-regulatory framework, allowing them to deal more effectively with sleep-interfering psychological processes and not perceive sleep disturbances as stressful as other races/ethnicities [26].

Prior research suggests that racial/ethnic disparities exist in the prevalence of symptoms among cancer survivors. In a survey of 139 BCSs, fatigue (76%) was the most commonly reported symptom followed by muscle aches (40%) [27]. Most BCSs complained of greater than six symptoms, and Hispanic women were more likely to report chemotherapy-related symptoms and pain-related symptoms compared to white BCSs. In another study of 199 cancer survivors, black cancer survivors experienced more pain interference, severity, and disability compared to whites [28]. Black BCSs were more likely to have chemotherapy and less likely to have surgery compared to whites. Pain associated with cancer may be associated with an increased morbidity and diminished quality of life for black women consistent with an unequal burden of cancer, pain, and cancer-related chronic pain [28].

Physical symptoms of pain and fatigue commonly cooccur with sleep disturbances. BCSs with fatigue showed significant differences in total sleep time (TST) and wake episodes compared to BCSs without fatigue [29]. Fatigued BCSs also reported worse QOL [30]. In addition, increased pain intensity over time significantly predicted sleep onset latency (SOL) in BCSs [31]. Sleep efficiency (SE) was also significantly lower for BCSs with pain [6]. The night before breast-conserving surgery, BCSs with low SE reported significantly more postoperative pain than BCSs with high SE.

Depression and anxiety are also frequently reported by BCSs. In one study [32] of 67 BCSs, depressed mood significantly predicted subjective sleep quality but not objective sleep in BCSs. Depressive symptoms also have been positively correlated with the prevalence and severity of subjective sleep in BCSs [33]. Anxiety has been correlated with subjective sleep [34], with worse sleep being significantly correlated with increased levels of anxiety in cancer patients. Previous work has demonstrated that black BCSs were less anxious in comparison to white BCSs [35]; this may be associated with higher social support, improved spirituality, and decreased fear of recurrence in black BCSs [36, 37].

We have limited insight on the effect of race/ethnicity on objective sleep disturbances (i.e., sleep actigraphy) and their association with physical and psychological symptoms. The existing gaps in our knowledge point to a need for additional research in this area. Therefore, the purposes of this study were to explore racial/ethnic differences in objective sleep disturbances among BCSs, explore racial/ethnic differences in the associations between objective sleep disturbances with symptoms, and explore the moderating effect of race/ethnicity on the relationship between objective sleep disturbances and symptoms. 

## 4. Method

### 4.1. Design and Sample

A cross-sectional research design was used to conduct a secondary data analysis of data from the National Cancer Institute (NCI) administrative sleep supplement study of the* MBSR Symptom Cluster Trial for Breast Cancer Survivors/*1R01CA131080. Within the larger study of 322 BCSs, a subsample of 79 completed baseline actigraphic monitoring. Inclusion criterions for the parent study were (1) female gender, age of 21 or older; (2) being diagnosed with stage 0, I, II, or III breast cancer; (3) having completed lumpectomy and/or mastectomy; (4) having from two weeks to two years from the end of treatment with adjuvant radiation and/or chemotherapy; (5) being able to read and speak English at the 8th grade level or above; and (6) having completed baseline actigraphic monitoring and self-reported questionnaires. Exclusion criteria consisted of (1) female gender, with advanced stage breast cancer (stage IV); (2) current diagnosis of severe current psychiatric disorder, and (3) having undergone recurrent treatment for prior breast cancer.

### 4.2. Setting

Participants were recruited from the H. Lee Moffitt Cancer Center and Research Institute and the Carol and Frank Morsani Center for Advanced Health Care located in Tampa, Florida. Participants enrolled in the study and completed baseline questionnaires in the Survivorship Clinic of the Moffitt Research Center.

### 4.3. Procedures

The study protocol was approved by the Institutional Review Board at the University of South Florida. A research assistant approached eligible BCSs prior to their scheduled appointment times; if patients were interested, they were invited to an orientation assessment where informed consent was obtained; they then completed baseline assessment of pain, fatigue, depression, and anxiety through self-reported questionnaires. After the completion of questionnaires, an actigraph was placed on the nondominant wrist of each participant. Participants were asked to continuously wear the actigraph for 72 hours and were provided with safety and care instructions. Participants were also provided with a prestamped, preaddressed envelope for the return of the actigraphy bracelet after 72 hours.

### 4.4. Instruments

All participants completed questionnaires presented in paper format. A demographic questionnaire queried participants' socioeconomic data including age, race/ethnicity, religion, education, marital status, employment status, and income. Participants completed a clinical history form for data including cancer diagnosis, cancer stage at time of diagnosis, type of breast cancer, length of time since cancer diagnosis, treatment type, and number of weeks on radiation/chemotherapy. Clinical history was verified by medical record review.

Objective sleep disturbances were measured by the Actiwatch Score actigraph (Philips Respironics, Andover, MA), which is a noninvasive method that uses internal motion sensors to capture patient movement data, represented as the number of accelerations per minute [38]. Spans without accelerometer movements reflect sleep. This data is then analyzed to produce a report containing parameters of sleep and wake periods, along with their timing, duration, and other characteristic details. Actigraphy scores correlated with EEG sleep/wake status 95% of the time and with polysomnography (PSG) within a range of ten percent [39].

Sleep disturbance was assessed using (1) SE, (2) SOL, (3) TST, and (4) wake after sleep onset (WASO). SE is defined as the percentage of time in bed spent sleeping [40]. It is calculated by dividing the number of minutes of sleep by the number of minutes in bed, multiplied by 100. SOL is the duration of time from bedtime to falling asleep [40]. TST is defined as the number of minutes of sleep while in bed [40]. WASO refers to the number of minutes awake after sleep onset during the sleep period [40]. All these parameters were measured in this study.

The Fatigue Symptom Inventory (FSI) is a 14-item self-report measure that is designed to assess severity, frequency, daily pattern of fatigue, and perceived interference with QOL [41]. Severity is measured on an 11-point scale (0: not at all; 10: extreme fatigue). Convergent validity and divergent validity were demonstrated with use of correlations with the fatigue scale of the Profile of Mood States Fatigue and the Short-Form General Health Survey (SF-36) vitality subscale [42]. The internal consistency reliability of the four-item FSI severity index in this study was alpha = 0.90.

Pain was measured by the Brief Pain Inventory (BPI) Short Form,which consists of 9 items that measure the severity of pain, impact of pain on daily function, location of pain, pain medications, and amount of pain relief in the past 24 hours or the past week [43]. The mean of the four severity items is used as a measure of pain severity, and the mean of the seven interference items is used as a measure of pain interference. The internal consistency reliability of the four-item BPI severity scale in this study was alpha = 0.94.

The Center for Epidemiological Studies Depression Scale (CES-D), used to measure depression, is a 20-item instrument that employs a 4-point scale (0: less than 1 day; 3: 5–7 days) to rate how frequently depressive symptoms were experienced during the past week [44]. Measures include cognitive, affective, behavioral, and somatic symptoms of depression and positive effect. The internal consistency reliability of the CES-D in this study was alpha = 0.91. Scores range from 0 to 60 with higher calculated scores indicative of more severe depressive symptoms.

Anxiety was measured with use of the State Trait Anxiety Inventory-State Scale only (STAI-S), a 20-item Likert scale that measures state anxiety ranging from 1 (almost never) to 4 (almost always) [45]. The STAI-S measures an individual's transitional emotional response, including worry, nervousness, tension, and feelings of apprehension to a stressful situation. The internal consistency reliability of the STAI-S in this study was alpha = 0.94. Total scores range from 20 to 80, with higher scores indicative of greater anxiety.

## 5. Data Analysis

Statistical Product and Services Solutions (SPSS) version 21.0 was utilized for all the deidentified data entry, data management, and analysis for this study. Frequency distributions and descriptive statistics were used to describe the sample demographic and clinical characteristics and actigraphy parameters. *t*-test and chi square analysis were used to compare sample demographic and clinical variables between the white, non-Hispanic, and the minority BCSs. Data analysis was conducted with a two-sided *P* value of less than 0.05 to define statistical significance. All demographic and clinical variables with a *P* value less than 0.05 were included as potential covariates for this analysis. Age was the only variable that was significantly (*P* = .041) different between the two groups and was included as a potential covariate for further analysis. Log transformation was completed to adjust for the skewness of data.

First, race/ethnicity was used to predict actigraphy parameters. The predictor of interest was race/ethnicity (i.e., white, non-Hispanic versus other races/ethnicities) and the defined outcome measures were SE, SOL, TST, and WASO. Analysis of variance (ANOVA) was used initially to compare unadjusted means of the defined outcome variables between the different racial groups, whereas analysis of covariance (ANCOVA) was used to compare adjusted means. The ANCOVA models were adjusted for age, which was a covariate that was imbalanced between the two groups. Secondly, to explore racial/ethnic differences in the associations between actigraphy parameters with physical and psychological symptoms, Pearson correlations were calculated for continuous variables and *t*-tests for variables with two categories. Significant correlations were further analyzed using multiple linear regression analysis with the actigraphy parameter as the dependent variable, race/ethnicity and symptoms as main effect terms, and an interaction term for physical or psychological symptom by race/ethnicity. Separate models were created for each actigraphy parameter. Simultaneous entry was used to enter independent variables in the regression model as the goal was to specifically explore the main effects of physical/psychological symptoms and race/ethnicity, respectively, compared to the interaction effect of physical/psychological symptoms with race/ethnicity on actigraphy parameters.

## 6. Findings

### 6.1. Sample Demographics

Of the 79 participants, one participant was a nightshift worker and her actigraphy data was not comparable to the sample and removed from the analysis; however, her remaining data was included. The racial/ethnic mix of the 79 participants was as follows: 60 (75.9%) white, non-Hispanic; 7 (8.9%) white, Hispanic; 8 (10.1%) black, non-Hispanic; 3 (3.8%) black, Hispanic; and 1 (1.3%) listed their ethnicity as other. The mean age of the sample was 55.5 (SD = 9.6) years with a mean age of 58.5 (SD = 8.6) years for white, non-Hispanic BCSs and 52.4 (SD = 11.3) years for minority BCSs.  Table 1 shows the treatment related factors for participants. The clinical variables listed in Table 1 were not statistically different by race/ethnicity (*P* > 0.05).  Table 2 displays the results summary of actigraphy parameters and physical and psychological symptoms. TST was significantly (*P* = 0.039) different between the groups with a greater percentage of minority BCSs experiencing TST outside the population norm [13] compared to the white, non-Hispanic group. Though not significantly different, more minority BCSs also experienced SOL and SE outside the population norm and reported higher anxiety, pain, and depression scores.

### 6.2. Racial/Ethnic Differences in Actigraphy Parameters

The means, standard deviations, mean differences, lower limit, upper limit, *P* values, and adjusted *P* values (with age as a covariate) of the actigraphy parameters for the white, non-Hispanic group were compared to those for the minority group (Table 3). Results suggest that TST was significantly higher for white, non-Hispanic participants (395.9 minutes) than for minority participants (330.4 minutes) (*P* = 0.01). In addition, minority participants took 35.7 minutes to fall asleep compared to 22.5 minutes for the white, non-Hispanic women (*P* = 0.07). Another nonsignificant trend for SE to be higher in white, non-Hispanic women (80%) compared to minority women (76%) (*P* = 0.09) was observed.

### 6.3. Relationship between Actigraphy Parameters and Symptoms

Correlations between the actigraphy parameters and subjective measures of depression (CES-D), fatigue (FSI), pain (BPI), and anxiety (STAI) for the white, non-Hispanic group were compared to those for the minority group (Table 4). Among minority BCSs, significant correlations were seen between SOL and depression (*r* = 0.453, *P* = 0.049), SOL and fatigue (*r* = 0.517, *P* = 0.028), and SE and fatigue (*r* = −0.535, *P* = 0.022). Among white, non-Hispanic BCSs, significant correlations were seen between SE and pain (*r* = −0.316, *P* = 0.014) and WASO and pain (*r* = 0.367, *P* = 0.040). No significant correlations were seen between anxiety and actigraphy parameters.

### 6.4. Race/Ethnicity, Actigraphy Parameters, and Symptoms

Table 5 displays the results of the multiple regression analyses for SOL with depression and fatigue, respectively, SE with fatigue and pain, respectively, and WASO with pain. In terms of main effects, pain (*P* = 0.017) had a significant effect on WASO. No main effects of depression or fatigue were found on actigraphy parameters. Race/ethnicity (*P* = 0.056) had a significant main effect on SE only. There was a significant interaction (*β* = 0.502, *P* = 0.046) between depression and race/ethnicity on their effect on SOL and a significant interaction (*β* = 0.596, *P* = 0.033) between fatigue and race/ethnicity on their effect on SOL.

## 7. Discussion 

To our knowledge, this study is the first to explore racial/ethnic differences in actigraphy parameters and the moderating effect of race/ethnicity on the association between actigraphy parameters and self-reported symptoms among BCSs. The current study yielded three main findings. First, actigraphy parameters indicated that white, non-Hispanic BCSs had better objective sleep compared to minority BCSs. Second, there was evidence of differential associations between symptoms and actigraphy parameters by race/ethnicity. Third, race/ethnicity modified the effect of depression and fatigue on SOL, respectively.

The first aim was to explore racial/ethnic differences in actigraphy parameters among BCSs. Of the actigraphy parameters, TST was significantly (*P* = 0.01) different between the groups with minority BCSs having a lower TST compared to white, non-Hispanic BCSs. Approximately 94% of the minority group had TST outside the accepted range compared to 63% of the white, non-Hispanic group. The National Sleep Foundation [46] poll indicated that blacks reported the least amount of sleep, averaging 38 min less sleep than whites. Lower levels of education, increased depressive symptoms, and higher body mass index were associated with short sleep durations of less than 420 minutes [47]. Recent evidence also suggests that residential environment may influence racial/ethnic differences in sleep duration [9].

In addition, there was a trend for SOL to be longer in minority BCSs compared to white, non-Hispanic BCSs. Though not significantly different, 61% of minority BCSs experienced prolonged SOL compared to 48% of white, non-Hispanic BCSs. Furthermore, a greater percentage of minority BCSs experienced decreased SE. These findings can be compared to previous studies that showed blacks slept for a shorter duration, took longer to fall asleep, and had lower sleep efficiency compared to whites [18, 48]. A recent meta-analysis also indicated similar findings with differences mediated by factors such as adiposity, mental illness, and employment status [49].

The second aim was to explore racial/ethnic differences in the relationships between actigraphy parameters with physical and psychological symptoms. Results suggest that these associations may differ between white, non-Hispanics and minorities. Specifically, depression was directly associated with SOL among minority BCSs; fatigue was directly associated with SOL and inversely associated with SE among minority BCSs; and pain was inversely associated with SE and directly associated with WASO among white BCSs only. Previous investigators evaluated relationships between symptoms and subjective sleep quality only. The magnitude of the correlations between sleep and fatigue, depression, pain, and anxiety ranged from 0.340 to 0.450, respectively, across seven studies [6, 50, 51]. Contrary to prior findings, TST was not associated with depression or fatigue [15].

The third aim was to explore the moderating effect of race/ethnicity on the relationships between actigraphy parameters and self-reported symptoms. There was no statistically significant effect of race/ethnicity, depression, or fatigue on SOL. However, the combination of depression by race/ethnicity and fatigue by race/ethnicity significantly predicted SOL suggesting that race/ethnicity modified the relationship of depression and fatigue on SOL. This indicates that minority BCSs who are depressed or fatigued may benefit from additional screening to determine if they are experiencing sleep disturbances. Fatigue, depression, and sleep disturbances are the most commonly reported symptoms by minority cancer survivors and often present together as symptom clusters in BCSs [12, 20, 52]. Special consideration should be given to minority BCSs who present with fatigue and depressive symptoms as these symptoms are often underrecognized and undertreated in minority BCSs [20, 53]. Minority cancer survivors showed a reluctance to use the word “depression,” making it more important for providers to assess depression in this population [54]. Several factors may contribute to increased depression in minority BCSs including increased financial barriers, lower social status, and decreased access to mental health services [53]. Cancer-related health worries are also a significant predictor of both depression and anxiety in black BCSs [55]. Early detection and assessment of these symptoms are essential to decrease sleep disturbances among BCSs.

Although race/ethnicity did not modify the effect of fatigue on SE and pain on SE and WASO, respectively, it was helpful in identifying symptoms that were perceived to contribute to sleep disturbances among BCSs. Pain had a significant main effect on WASO and an insignificant main effect on SE. A previous study reported a strong correlation between SE and pain severity among BCSs [6]. Consistent with previous findings, pain was most commonly associated with sleep disturbances among BCSs [56]. Race/ethnicity also had a significant main effect on SE. Past research has indicated that blacks have poorer sleep efficiency compared to whites [16, 49].

### 7.1. Limitations

Due to the small sample size and imbalanced number of racial/ethnic participants, there may be an underestimation of the strength of a true statistical association (i.e., type II error) or alternatively an overestimation of the effect size and the possibility of type I error. Second, adjustments for multiple comparisons were not completed due to the exploratory nature of the study. Third, the cross-sectional design of the study did not allow for causal relationships between variables to be determined. Of the 79 participants, there were 19 minority patients in the study which represented both blacks and Hispanics. These two groups may differ in regard to sleep patterns. Future studies should incorporate larger samples of blacks and Hispanics with an attempt to look at differences in sleep patterns between these two groups. Finally, factors that may be associated with differences in actigraphy parameters between races/ethnicities should also be considered. These include demographic risk factors (educational status, employment status, and marital status), clinical factors (medical comorbidities, medications used to treat comorbidities, and the use of sleeping pills), and lifestyle factors (alcohol/cigarette consumption, physical activity, and social support) [15].

### 7.2. Implications for Research

In addition to exploring changes in sleep disturbances between racial/ethnic groups, the findings of this study may facilitate the development of culturally appropriate, survivorship plans. The National Comprehensive Cancer Network has recently developed guidelines for survivorship care, specifically addressing sleep disorders in cancer survivors. These guidelines can only be generalizable if research is further conducted on minority BCSs. Specifically, longitudinal studies should explore the effect of physical symptoms, psychological symptoms, and race/ethnicity on sleep disturbances. Without such studies, survivorship care plans will be applicable to the white, non-Hispanic population.

This study has several implications for research. Future studies incorporating larger sample sizes with equal representation of white, non-Hispanic BCSs and minority BCSs are needed. In addition, our findings indicate that the relationship between symptoms and sleep disturbances are different by races/ethnicities. Results suggest that depression and fatigue are significant predictors of sleep disturbances among minority BCSs and pain is a significant predictor of sleep disturbances in white, non-Hispanic BCSs. Additional research is needed to evaluate these relationships. Time since completion of treatment did not differ by race/ethnicity, yet this may be an important variable to be further examined in relation to sleep disturbances in BCSs.

Furthermore, longitudinal studies are needed to examine the physical, emotional, and psychosocial contributing factors to shortened TST in minority BCSs compared to white, non-Hispanic BCSs [21]. Fewer hours of sleep has been associated with higher recurrence scores and more aggressive tumors in BCSs [57]. The association of shortened TST and aggressive tumors in minority BCSs should also be further explored. Longitudinal studies implementing longer periods of wrist actigraphy are also required in this population [58]. This study focused on BCSs who remained disease-free after treatment; it is essential that future research also incorporate minority BCSs with recurrent and advanced cancer.

### 7.3. Implications for Nurses

The subjective and objective assessment of sleep disturbances as part of survivorship care for BCSs is ideal; however, objective assessments may not be feasible in a clinical setting, indicating that practice guidelines assessing sleep are essential. The National Comprehensive Cancer Network (NCCN) guidelines for sleep disorders recommend screening cancer survivors at regular intervals [59]. The following questions are recommended for screening: “do you have difficulty falling or staying asleep,” “how long does it take to fall asleep,” “how many times do you wake up every night,” or “how long have you had difficulty in falling or staying asleep?” If indicated after screening, nurses should conduct comprehensive diagnostic exams including a sleep-oriented history, physical examination, objective testing, and education [60]. Routine assessment of sleep duration should be completed at regular physical exams, specifically for minority BCSs [23]. Inquiring about TST, by asking patients “how many hours are you sleeping at night,” may be more helpful in the identification of BCSs who are at a higher risk of sleep disturbances. Assessments of depression, fatigue, and pain should also be incorporated as part of routine sleep assessment. Culturally appropriate questions during follow-up visits may reduce response bias associated with self-reports, particularly when assessing sleep disturbances and depression in black BCSs [23].

Special consideration should be placed on sleep behaviors among BCSs of different races/ethnicities with attention to sleep hygiene practices, sleep partners, and use of sleep medications. Blacks spend more time in bed without sleeping compared to other ethnic groups and are more likely to report performing activities in the hour before going to bed such as watching TV or praying [46]. Alerting bedtime routines may contribute to increased SOL among minority BCSs. Secondly, a sleep partner could be a contributing factor to sleep disturbances. Asians and Hispanics are more likely to sleep in the same room with their children; whites are more likely to sleep with their significant other; and blacks are most likely to sleep alone [46]. Finally, the use of sleep medications should be considered. Asians are least likely to take sleep medications; whites are more likely to use over-the-counter sleep aids; and blacks are more likely to take prescribed medications.

Nurses are in an ideal position to assess, educate, develop, and integrate evidence-based practice into the management of sleep disturbances among BCSs. Clinical sleep assessments should be a part of comprehensive assessment tools and should be personalized towards minority BCSs. Education provides nurses with the opportunity to teach BCSs about techniques and interventions to manage sleep disturbances. A previous study [61] documented that both black and Hispanic women were not aware of resources for their sleeping problems. Hence, BCSs with sleep disturbances should be informed about interventions for the management of their symptoms and should be encouraged to participate in such interventions, which have been beneficial in addressing symptoms associated with sleep disturbances, including pain, fatigue, and depression [12]. In addition, oncology nurses must collaboratively work with minority BCSs and their families to incorporate sleep hygiene practices and generate shared goals to improve sleep quality in BCSs. Financial barriers, residential and social status, and access to follow-up care should be addressed as these factors may contribute to increased depression and sleep disturbances in minority BCSs [9, 53]. As required, referral to sleep specialists can also be made [9, 59].

## 8. Conclusion

The study's findings suggest that minority BCSs have shorter sleep durations compared to white, non-Hispanic BCSs. In addition, pain, fatigue, and depression may be important covariates of sleep disturbances in BCSs. We hypothesize that race/ethnicity modified the association between physical/psychological symptoms and sleep disturbances. As part of survivorship care, race/ethnicity should be included as an essential component of comprehensive symptom assessments in BCSs. Comprehensive sleep assessments including appropriate assessments of pain, fatigue, and depression should be considered when developing and implementing patient centered, culturally appropriate interventions.

## Figures and Tables

**Table 1:** Surgery type, treatment type, stage of cancer, type of breast cancer, and time since completion of treatment of participants by race/ethnicity (*N* = 79).

Variable	Total	White, non-Hispanic	Minority	*P* value
	*n* = 79	*n* = 60	*n* = 19	
Surgery type, *n* (%)				0.662
Lumpectomy	34 (43.1)	25 (41.7)	9 (47.4)	
Mastectomy	45 (56.9)	35 (58.3)	10 (52.6)	
Treatment type, *n* (%)				0.403
Chemotherapy	10 (12.7)	7 (11.7)	3 (15.8)	
Radiation	21 (26.6)	15 (25.0)	6 (31.6)	
Chemotherapy and radiation	23 (29.1)	16 (26.7)	7 (36.8)	
Surgery only	25 (31.6)	22 (36.7)	3 (15.8)	
Cancer stage, *n* (%)				0.854
0	13 (16.5)	9 (15.0)	4 (21.1)	
1	27 (34.2)	21 (35.0)	6 (31.6)	
2	27 (34.2)	20 (33.3)	7 (36.8)	
3	12 (15.2)	10 (16.7)	2 (10.5)	
Type of breast cancer, *n* (%)				0.416
Ductal carcinoma in situ	13 (16.5)	9 (15.0)	4 (21.1)	
Invasive lobular	26 (32.9)	20 (33.3)	6 (31.6)	
Invasive ductal	22 (27.8)	18 (30.0)	4 (21.1)	
Not specified	3 (3.8)	1 (1.7)	2 (10.5)	
Unknown	15 (19.0)	12 (20.0)	3 (15.7)	
Time since completion of treatment, *n* (%)				0.462
2 weeks to 1 year	42 (53.2)	31 (51.7)	11 (57.9)	
1 year to 2 years	37 (46.8)	29 (48.3)	8 (42.1)	

**Table 2:** Descriptive statistics of actigraphy parameters, physical and psychological symptoms of participants by race/ethnicity (*N* = 78).

Variables	Minimum	Maximum	Mean	SD	Population normal	Percentage abnormal (white)	Percentage abnormal (minority)	*P* value
TST	234	582	380.8	62.64	420–540 [13]^a^	63.33	94.44	0.039∗
SOL	0	122	25.57	26.13	<20 [13]^a^	48.33	61.11	0.714
SE	56	91	78.76	7.66	>85 [13]^a^	76.67	83.33	0.301
WASO	11	144	61.97	28.11	<42 [13]^a^	83.33	66.67	0.267
STAI	20	78	37.55	11.45	<32.2 [45]^b^	56.67	77.78	0.365
BPI	0	8.75	2.54	2.28	<7 [43]^c^	5.00	11.11	0.187
FSI	0	9	4.03	1.89	≤3 [41]^d^	60.00	38.89	0.922
CES-D	0	55	14.94	10.41	<16 [44]^e^	36.67	38.89	0.863

BPI: Brief Pain Inventory Short Form Severity Scale; CES-D: Center for Epidemiological Studies Depression Scale; FSI: Fatigue Symptom Inventory Severity Scale; SE: sleep efficiency; SOL, sleep onset latency; STAI-S: State Trait Anxiety Inventory-State Scale only; TST: total sleep time; WASO: wake after sleep onset.

Data from the ^a^American Academy of Sleep Medicine [13]; ^b^Spielberger et al. [45]; ^c^Cleeland [43]; ^d^Hann et al. [41]; ^e^Radloff [44].

∗Variable is significant at the 0.05 level.

**Table 3:** Means, standard deviations, mean differences, lower and upper limits, and significance of actigraphy parameters of participants by race/ethnicity (*N* = 78).

Actigraphy parameter	Group	*n*	Mean	Standard deviation	Mean difference	Lower limit	Upper limit	*P* value	Adjusted *P* value
TST	Minority	18	330.39	57.47	−65.53	301.81	358.97	0.01	0.01∗
	White	60	395.92	56.22	−65.53	381.39	410.44		

SOL	Minority	18	35.69	31.61	13.15	19.97	51.44	0.12	0.07
	White	60	22.53	23.71	13.15	16.41	28.66		

SE	Minority	18	75.93	8.59	−3.68	71.66	80.21	0.07	0.09
	White	60	79.61	7.22	−3.68	77.75	81.48		

WASO	Minority	18	56.22	26.19	−7.48	43.21	69.25	0.33	0.39
	White	60	63.69	28.65	−7.48	56.33	71.11		

SE: sleep efficiency; SOL: sleep onset latency; TST: total sleep time; WASO: wake after sleep onset.

∗Actigraphy parameter is significant at the 0.05 level.

**Table 4:** Correlations between sleep actigraphy parameters and depression, fatigue, pain, and anxiety (*N* = 78).

Actigraphy parameter	Group	*n*	Depression	Fatigue	Pain	Anxiety
TST	Minority	18	0.014	−0.277	0.236	0.423
	White	60	0.013	−0.129	0.065	0.029

SOL	Minority	18	0.453∗	0.517∗	0.203	0.281
	White	60	0.160	0.243	0.114	0.081

SE	Minority	18	−0.361	−0.535∗	−0.092	−0.107
	White	60	−0.178	−0.106	−0.316∗	−0.066

WASO	Minority	18	0.273	0.340	0.048	0.199
	White	60	0.163	0.111	0.367∗	0.143

SE: sleep efficiency; SOL: sleep onset latency; TST: total sleep time; WASO: wake after sleep onset.

∗Correlations are significant at the 0.05 level.

**Table 5:** Multiple regression analysis of race/ethnicity and symptoms in relation to sleep actigraphy parameters (*N* = 78).

Dependent variable	Primary predictors	Unstandardized coefficient		Standardized coefficients	*T*	*P* value	Adjusted *R* squared
		*b*	Std. error	*β*			
SOL (minutes)	Race/ethnicity	−2.72	5.22	−0.091	−0.522	0.603	
	Depression	−0.262	0.505	−0.104	−0.519	0.605	
	Depression by race/ethnicity	0.617	0.304	0.502	2.03	0.046∗	
							0.110

SOL (minutes)	Race/ethnicity	−6.44	6.65	−0.216	−0.968	0.336	
	Fatigue	−2.69	2.81	−0.195	−0.959	0.341	
	Fatigue by race/ethnicity	3.39	1.57	0.596	2.17	0.033∗	
							0.090

SE (percent)	Race/ethnicity	0.722	1.94	0.082	0.372	0.711	
	Fatigue	0.280	0.819	0.069	0.342	0.733	
	Fatigue by race/ethnicity	−0.774	0.456	−0.463	−1.70	0.094	
							0.101

SE (percent)	Race/ethnicity	−2.62	1.35	−0.299	−1.94	0.056∗	
	Pain	−1.14	0.717	−0.339	−1.59	0.117	
	Pain by race/ethnicity	−0.298	0.410	0.177	0.727	0.469	
							0.064

WASO (minutes)	Race/ethnicity	2.89	4.95	0.090	0.585	0.560	
	Pain	6.43	2.64	0.522	2.44	0.017∗	
	Pain by race/ethnicity	−1.98	1.51	−0.322	−1.32	0.192	
							0.064

SE: sleep efficiency; SOL: sleep-onset latency; WASO: wake after sleep onset.

∗Correlations are significant at the 0.05 level.
